# Exposure to family and domestic violence is associated with increased childhood hospitalisations

**DOI:** 10.1371/journal.pone.0237251

**Published:** 2020-08-07

**Authors:** Carol Orr, Colleen M. Fisher, David B. Preen, Rebecca A. Glauert, Melissa O’Donnell

**Affiliations:** 1 School of Population and Global Health, The University of Western Australia, Perth, Western Australia, Australia; 2 Telethon Kids Institute, The University of Western Australia, Perth, Western Australia, Australia; Ravensburg-Weingarten University of Applied Sciences, GERMANY

## Abstract

**Background:**

Children’s exposure to family and domestic violence (FDV) is a global public health concern and is considered one of the most common and severe stressors children can experience. While it is acknowledged that children who are exposed to FDV have poorer general health, there is a lack of data on the outcomes of children exposed to FDV. The use of longitudinal data has been suggested as a way to gain an understanding of the impact on children’s long-term outcomes.

**Methods:**

Our cohort study used deidentified individual-level linked administrative data of children born 1987–2010, in Western Australia, who were exposed to FDV in the prenatal period (12 months prior to birth) to five years of age (early years).

**Results:**

Children exposed to FDV are more likely to be hospitalised than non-exposed children. Children exposed to FDV in both the prenatal and early childhood period had a threefold increased odds of mental health hospitalisation. We found a significant increase in odds of pregnancy-related hospitalisation in FDV exposed children. When stratified by Aboriginal status, Aboriginal children had a higher proportion of hospitalisations than non-Aboriginal children.

**Conclusion:**

Exposed children have an increased likelihood for hospitalisation than non-exposed children. Within the exposed cohort differences were apparent between Aboriginal and non-Aboriginal children. Aboriginal children had greater odds for hospitalisation in most of the diagnostic groups compared to their non-Aboriginal counterparts. Our findings represent an important advance in the literature with respect to the burden of disease of children exposed to FDV.

## Introduction

Children’s exposure to family and domestic violence (FDV) is a global public health concern. It has been estimated that 275 million children worldwide are exposed to FDV [1], however, due to its hidden nature it is accepted that this figure is a conservative estimate with millions more children likely affected. The term ‘family and domestic violence’ is used in Australia to encompass acts including intimate partner violence, abuse between siblings and other family members and between extended kinship ties. While the term FDV is encompassing it should not detract from the fact that women are disproportionately affected by violence perpetrated by men [2, 3]. Within Australia, FDV perpetrated by a current or former intimate partner is a widespread issue with a lifetime prevalence of 25% [4]. Almost two-thirds of female victims of FDV in Australia are assaulted in their own home, with 49% of all female victims having children in their care at the time of the assault [4]. Children’s exposure to FDV can be defined as witnessing and hearing acts first hand, as well as the effects of living with the consequences of the incident(s), such as seeing their mothers distressed or with physical injuries, damaged furniture, and homelessness [5, 6]. Pre-school children are particularly vulnerable and disproportionately exposed to FDV due to the time they spend in the family home [7–9]. As a result of the legacy of colonisation, Aboriginal and Torres Strait Islander children (hereafter respectfully referred to as Aboriginal) are also at greater risk of exposure due to the higher rates of FDV within Aboriginal populations when compared to non-Aboriginal Australians [10, 11]. While the outcomes for women who are victims of FDV are well researched, the literature is lacking on the outcomes for children who are exposed, with children being referred to as the forgotten victims [12].

Evidence from the limited studies undertaken, have found that children who are exposed to FDV are more likely to have poorer general physical health than the general population of children [13], experience gastrointestinal problems [14], and psychological health issues [8, 15, 16] at higher rates than non-exposed children. The biological mechanisms linking stress to ill health are not fully understood, however, considerable evidence points to a central role of the stress axes, the hypothalamic-pituitary-adrenal-axis (HPA-axis) [17]. From a biological perspective, the stress caused by FDV exposure can challenge the physiological response systems, particularly the HPA-axis [18].

The Australian Institute of Health and Welfare report [19] highlighted the lack of data on children exposed to FDV and acknowledged that such children are an at-risk group, and comprehensive data are required to determine how FDV exposure affects their lives and health in the longer term. The report recommends the use of longitudinal data and multiple data sources, such as police, to gain a full understanding of the impact of FDV on children. Using longitudinal linked administrative data, we hypothesise that FDV exposure between the prenatal period (12 months prior to birth) and five years of age will be associated with an increase in childhood hospitalisation, compared to non-exposed children. We further expect to see increased levels of vulnerability of hospitalisation for specific health areas such as mental health and gastrointestinal problems.

## Methods

### Data sources

This retrospective cohort study used administrative datasets which were linked by the WA Data Linkage Branch [20] using probabilistic matching and clerical review [21]. Previous studies have found linkage accuracy to be above 99% [22, 23]. The researchers were provided de-identified information from the administrative datasets with linkage keys for each cohort member and their mother [24]. Using the linkage keys, the deidentified data were merged across six datasets: WA Police (2004–2008), WA Register of Developmental Anomalies (1987–2016), the Intellectual Disability Exploring Answers database (1987–2016), Birth Registration (1987–2010), Midwives Notification System (1987–2010) and Hospital Morbidity Data Collection (1979–2016).

Aboriginal children were identified by the WA Data Linkage Branch derived Aboriginal status flag. The flag is created by a validated algorithm when an individual is recorded as Aboriginal in WA government administrative data sets [25]. Neighbourhood-level socioeconomic status (SES) was determined by the Socio-Economic Indexes for Areas (SEIFA) using the midwives’ data. The SEIFA score is based on information about income, education, employment, occupation and housing; providing a measure of relative socio-economic status about the area where a person resides. Five levels of disadvantage were assigned to census collection districts (~250 households), ranging from 1 (high disadvantage) to 5 (low disadvantage) [26]. Residential remoteness was determined by the Accessibility/Remoteness Index of Australia (ARIA) which are based on distance of geographic locations from the nearest population centre criteria ranging from major cities to very remote Australia [27]. Residential remoteness was identified in the midwives data from collection district, the smallest spatial unit available [28].

### Ethics

To access the de-identified administrative data, the researchers engaged with Data Custodians and relevant stakeholders during the rigorous application process [20]. During the linkage process ‘separation principle’ [29], was adhered to by the WA Data Linkage Branch to safeguard privacy, this principle is used internationally and accepted as ‘best practice’ [20]. Ethics approval for this study was obtained from the WA Department of Health Human Research Ethics Committee (#2016/60), the WA Aboriginal Health Ethics Committee (#756), and the University of Western Australia Human Research Ethics Committee (#RA/4/1/8867).

### Definition of FDV exposure

The term exposed to FDV is used in this study as it is inclusive of the different types of experiences of the child and does not assume that the child observed the FDV. Children’s exposure to FDV includes witnessing and hearing acts first hand, as well as the effects of living in the aftermath of the incident(s) (for a taxonomy of exposure see Holden [30].

### Identification of FDV exposure in the study population

Exposure to FDV was initially identified in children born from 1987–2010 whose mother was identified in WA Police data between 2004 and 2008 as a victim of FDV where a male perpetrator was charged for the offence and a domestic relationship indicator was present. The criminal offences were: murder, attempted murder, physical assault, sexual assault, threatening behaviour, and misuse of weapons. There were 15,598 children born to mothers who were identified as victims of FDV in police data. The children were matched 1:3 (15,598: 46,438) to a non-FDV exposed cohort of children based on month of birth, socioeconomic status, Aboriginal status, and sex. Due to the hidden nature of FDV we also examined non-exposed children for any maternal FDV hospitalisations by interrogating the hospital records of their mothers from 1979 to 2016 using International Classification of Diseases (ICD) Codes [31] described elsewhere [32]. We found that 4,442 ‘non-exposed’ children had a mother who had a hospitalisation for FDV. These children were then classified as exposed to FDV. We then restricted the exposure period to violence that occurred between the prenatal period (12 months prior to birth) and the early years (birth to 5 years of age), which resulted in an exposed cohort of 7,957 and a non-exposed comparison group of 41,996. Exposure was identified using available data, given the prevalence of FDV in Australia there will be children exposed in the ‘non-exposed’ group who we have been unable to identify.

### Timing of FDV exposure and hospitalisation

Exposure to FDV could occur in the prenatal period or at any point in the first five years of the child’s life with hospitalisations counted at any point from birth. While it is possible, for the early years group, that a hospitalisation preceded FDV exposure recorded in our data; this approach was taken because it is recognised that for many women FDV is not a one-off occurrence [33–35]. Therefore, it is unlikely that the available data captures the first incident of FDV. Many women remain silent about their abuse or if they do seek help it may be years after the first incident [4, 36–38]. Women’s help-seeking is affected by multiple factors: stigma, fear, lack of a safe place to disclose [39, 40], feeling they can deal with it themselves, or believing it is not serious enough to seek help [4] and fearful of their children being removed [37, 38].

### Children’s hospitalisation diagnosis

The principal diagnosis for each hospitalisation is documented on hospital discharge forms. Hospital discharges prior to July 1999 were coded using the Ninth revision of ICD with clinical modifications (ICD-9-CM) [41]. Discharges from July 1999 to December 2016 (end of follow up) were classified using the Tenth Revision ICD with Australian modifications (ICD-10-AM). ICD-9-CM discharges were mapped to their equivalent ICD-10-AM code [42]. There are 17 major diagnostic groups in ICD-10-AM and three additional categories covering external cause of injury and unlisted signs and symptoms.

### Analysis

Multivariate logistic regression analysis was used to calculate odds ratios and 95% confidence intervals to determine the association with hospitalisations for children exposed to FDV compared to non-exposed children. While there is some debate within literature in the utility of p-values [43, 44] we have provided nominal p-values which have not been corrected for multiple-hypothesis testing [45]. It has been argued that while p-value adjustment may reduce the chance of making a type I error, it can also increase the chance of making a type II error [45, 46]. Type II errors are no less important than type I errors [47]. Therefore, for this study p-values were not adjusted. Model adjustment was made for sex, socioeconomic status, Aboriginal status, mothers’ maternal age, mothers’ marital status, gestational age, disability, birth year, and residential remoteness. All analyses were conducted using SAS® statistical software V9.4 [48]. Analysis was carried out separately for children exposed in the prenatal period and children exposed in the early childhood period (birth to 5 years of age). Analysis for children exposed in early childhood was also stratified by Aboriginal status. Firth bias-correction was applied where quasi-complete separation occurred, which uses a penalised likelihood estimation method considered as an ideal solution to separation issue for logistic regression [49]. Specific reasons for hospitalisations within ICD groupings of ‘pregnancy, childbirth and the puerperium’ and ‘mental and behavioural disorders’ are provided as proportions.

## Results

### Demographics

There were 12.4% more Aboriginal children in our exposed cohort compared with non-exposed group. Compared to the non-exposed group the exposed group were 5.2% more likely to have been born <37 weeks gestation and have a young mother (<20 years). Further demographics of the exposed and non-exposed children are available in Table 1. The differences between the two groups are accounted for in subsequent analyses.

**Table 1:** Demographic characteristics of the study cohort.

Attribute	FDV exposed (n = 7,957)	Non-Exposed (n = 41,996)
sex		
female	3960 (49.8)	20537 (48.9)
male	3997 (50.2)	21459 (51.1)
Aboriginal		
yes	4951 (62.2)	20919 (49.8)
No	3006 (37.8)	21077 (50.2)
Maternal age		
<20	1640 (20.6)	6122 (14.6)
20–29	4,609 (57.9)	22774 (54.2)
30–39	1,630 (20.5)	12478 (29.7)
40+	78 (1)	611 (1.5)
Gestation age		
< 37 weeks	1241 (15.6)	4353 (10.4)
Mothers marital status		
Married/defacto/widowed	5371 (67.5)	33300 (79.3)
Never married	2269 (28.5)	7920 (18.9)
Divorced/separated	228 (2.9)	564 (1.3)
Unknown	89 (1.1)	212 (0.5)
Socioeconomic status		
1—*Most disadvantaged*	4185 (52.6)	20569 (49)
2	1689 (21.2)	9549 (22.7)
3	1197 (15.0)	6251 (14.9)
4	601 (7.6)	3867 (9.2)
5—*Least disadvantaged*	285 (3.6)	1760 (4.2)
Residential remoteness		
1—*Highly accessible*	3430 (43.1)	20261 (48.3)
2	779 (9.8)	4520 (10.8)
3	1145 (14.4)	6725 (16.0)
4	1024 (12.9)	5190 (12.4)
5—*Very remote*	1579 (19.8)	5300 (12.6)
Child disability		
Yes	710 (8.9)	3061 (7.3)
No	7247 (91.1)	38935 (92.7)
Birth year		
1987–1992	217 (2.7)	6419 (15.3)
1993–1998	631 (7.9)	9322 (22.2)
1999–2004	3539 (44.5)	13179 (31.4)
2005–2010	3570 (44.9)	13076 (31.1)

### Children exposed to FDV in the prenatal period

There were 1,477 children who were exposed to FDV in the prenatal period (Table 2), accounting for 18.6% of our exposed cohort. Following adjustment for sociodemographic factors, these children had a 40% increased odds of hospitalisation compared with children who were not exposed in the prenatal period (OR 1.40; 95% CI 1.20–1.62). Compared to the non-exposed children, exposed children had a threefold increase in odds for mental health hospitalisation (OR 3.16; 95% CI 2.11–4.75) and hospitalisation for their own subsequent pregnancy (OR 2.97; 95% CI 1.80–4.91). Significant increased odds were also seen for hospitalisations due to infectious disease (OR 1.62; 95% CI 1.42–1.86), dermatological issues (OR 1.56; 95% CI 1.33–1.83), respiratory system problems (OR 1.49; 95% CI 1.33–1.67), and ear disorders (OR 1.35; 95% CI 1.14–1.60).

**Table 2:** Adjusted* principal hospitalisations for children exposed and non-exposed to FDV in prenatal period.

ICD group		Violence n = 1,477 (%)	No violence n = 41,996(%)	OR (95%CI)	P-value
	Any hospitalisation <18 years	1,240 (84)	33,977 (80.9)	1.40 (1.20–1.62)	<0.001
A00-B99	Infectious disorders	326 (22.1)	6,529 (15.6)	1.62 (1.42–1.86)	<0.001
C00-D48	Neoplasms	12 (0.8)	575 (1.4)	0.97 (0.54–1.75)	0.916
D50- D89	Diseases of the blood	7 (0.5)	325 (0.8)	0.60 (0.28–1.29)	0.193
E00-E89	Endocrinology disorders	28 (1.9)	719 (1.7)	1.15 (0.77–1.72)	0.493
F00-F99	Mental and behavioural disorders	29 (2)	684 (1.6)	3.16 (2.11–4.75)	<0.001
G00-G99	Diseases of the nervous system	56 (3.8)	1,598 (3.8)	0.81 (0.61–1.07)	0.140
H00-H59	Eye disorders	37 (2.5)	890 (2.1)	1.31 (0.92–1.85)	0.131
H60-H99	Ear disorders	176 (11.9)	3,902 (9.3)	1.35 (1.14–1.60)	0.001
I00-I99	Diseases of the circulatory system	23 (1.6)	595 (1.4)	1.13 (0.73–1.75)	0.575
J00-J99	Respiratory disorders	547 (37.0)	12,272 (29.2)	1.49 (1.33–1.67)	<0.001
K00-K93	Digestive disorders	221 (15)	7,814 (18.6)	1.00 (0.86–1.16)	0.955
L00-L99	Dermatology disorders	212 (14.4)	3,849 (9.2)	1.56 (1.33–1.83)	<0.001
M00-M99	Musculoskeletal disorders	50 (3.4)	1,466 (3.5)	1.23 (0.91–1.65)	0.181
N00-N99	Disease of genitourinary system	96 (6.5)	2,717 (6.5)	1.25 (1.0–1.55)	0.050
O00-O99	Pregnancy, childbirth and the puerperium ^	22 (1.5)	964 (2.3)	2.97 (1.80–4.91	<0.001
P00-P96	Certain conditions originating in the perinatal period	560 (37.9)	12,109 (28.8)	1.12 (0.98–1.26)	0.088
Q00-Q99	Congenital malformations, deformations and chromosomal abnormalities	102 (6.9)	2,290 (5.5)	1.17 (0.92–1.49)	0.204
R00-R99	Symptoms, signs and abnormal clinical and laboratory findings not elsewhere classified	240 (16.3)	5,151 (12.3)	1.51 (1.30–1.75)	<0.001
S00-T98	Injury and poisoning (and certain other consequences of external causes)	363 (24.6)	9,261 (22.1)	1.42 (1.25–1.61)	<0.001
Z00-Z99	Factors influencing health status and contact with health services	245 (16.6)	7,796 (18.6)	1.31 (1.13–1.52)	<0.001

*Adjusted for sex, socioeconomic status, Aboriginal status, mothers maternal age, mother’s marital status, gestational age, disability, birth year, and remoteness.

^ Firth bias correction applied due to quasi-complete separation caused by birth year.

OR = Odds ratio, CI = Confidence Interval.

Within the additional ICD-10-AM categories, compared to their non-exposed counterparts, FDV-exposed children had 51% greater odds to have a hospitalisation for symptoms not classified in the ICD-10-AM major category groups (OR 1.51; 95% CI 1.30–1.75). Similarly, FDV exposed children had an increased odds of being hospitalised for injury and poisoning (OR 1.42; 95% CI 1.25–1.61), and for external factors influencing health status (OR 1.31; 95% CI 1.13–1.52) compared with non-exposed children.

### Children exposed in the early years

There were 7,018 children exposed to FDV in the early years (birth to 5-years) category (Table 3). Compared to the non-exposed children these children had a 34% greater odds of hospitalisation (OR 1.34; 95% CI 1.23–1.43). Similar to the children exposed in the prenatal period the children exposed to FDV in the early years had much greater odds for mental health hospitalisation (OR 2.65; 95% CI 2.19–3.21) compared to their non-exposed counterparts. The children exposed to FDV had a twofold increased odds of hospitalisation for their own subsequent pregnancy (OR 1.93; 95% CI 1.55–2.40) compared to the non-exposed children. Increased odds for exposed children were also seen in endocrinology disorders (OR 1.73; 95% CI 1.46–2.03), infectious disease (OR 1.63; 95% CI 1.52–1.74), and dermatology disorders (OR 1.59; 95% CI 1.47–1.72). Further increased odds were found in the ICD categories of eye disorders (OR 1.41; 95% CI 1.20–1.67) and ear and respiratory system problems (OR 1.37; 95% CI 1.26–1.49; OR 1.37; 95% CI 1.29–1.45 respectively). Additionally, the children had higher odds of external factors influencing health status (OR 1.25; 95% CI 1.16–1.35), and injury and poisoning (OR 1.48; 95% CI 1.39–1.58).

**Table 3:** Adjusted* principal hospitalisations for children exposed and non-exposed to FDV in early childhood period.

ICD group	All adjusted	Violence n = 7,018 (%)	No violence n = 41,996(%)	OR (95%CI)	P-value
	Any hospitalisation <18 years	5,960 (84.9)	33,977 (80.9)	1.34 (1.23–1.43)	<0.001
A00-B99	Infectious disorders	1,713 (24.4)	6,529 (15.6)	1.63 (1.52–1.74)	<0.001
C00-D48	Neoplasms	70 (1.0)	575 (1.4)	0.93 (0.72–1.21)	0.596
D50- D89	Diseases of the blood	52 (0.7)	325 (0.8)	0.95 (0.70–1.29)	0.726
E00-E89	Endocrinology disorders	201 (2.9)	719 (1.7)	1.73 (1.46–2.06)	<0.001
F00-F99	Mental and behavioural disorders	164 (2.3)	684 (1.6)	2.65 (2.19–3.21)	<0.001
G00-G99	Diseases of the nervous system	278 (4.0)	1,598 (3.8)	0.92 (0.80–1.05)	0.210
H00-H59	Eye disorders	200 (2.85)	890 (2.1)	1.41 (1.20–1.67)	<0.001
H60-H99	Ear disorders	890 (12.68)	3,902 (9.3)	1.37 (1.26–1.49)	<0.001
I00-I99	Diseases of the circulatory system	145 (2.07)	595 (1.4)	1.33 (1.10–1.62)	0.004
J00-J99	Respiratory disorders	2,601 (37.06)	12,272 (29.2)	1.37 (1.29–1.45)	<0.001
K00-K93	Digestive disorders	1,194 (17.01)	7,814 (18.6)	1.02 (0.95–1.91)	0.673
L00-L99	Dermatology disorders	1,062 (15.13)	3,849 (9.2)	1.59 (1.47–1.72)	<0.001
M00-M99	Muscular skeletal disorders	260 (3.70)	1,466 (3.5)	1.16 (1.01–1.34)	0.042
N00-N99	Disease of genitourinary system	469 (6.68)	2,717 (6.5)	1.09 (0.98–1.21)	0.132
O00-O99	Pregnancy, childbirth and the puerperium ^	128 (1.82)	964 (2.3)	1.93 (1.55–2.40)	<0.001
P00-P96	Certain conditions originating in the perinatal period	2,581 (36.78)	12,109 (28.8)	1.10 (1.03–1.17)	0.002
Q00-Q99	Congenital malformations, deformations and chromosomal abnormalities	404 (5.76)	2,290 (5.5)	0.95 (0.83–1.08)	0.430
R00-R99	Symptoms, signs and abnormal clinical and laboratory findings not elsewhere classified	1,138 (16.22)	5,151 (12.3)	1.43 (1.32–1.54)	<0.001
S00-T98	Injury and poisoning (and certain other consequences of external causes)	1,978 (28.18)	9,261 (22.1)	1.48 (1.39–1.58)	<0.001
Z00-Z99	Factors influencing health status and contact with health services	1,195 (17.03)	7,796 (18.6)	1.25 (1.16–1.35)	<0.001

*Adjusted for sex, socioeconomic status, Aboriginal status, mothers maternal age, mother’s marital status, gestational age, disability, birth year, and remoteness.

^ Firth bias correction applied due to quasi-complete separation caused by birth year.

OR = Odds ratio, CI = Confidence Interval.

### Mental health hospitalisations

Mental health admissions for both prenatal and early childhood exposed groups were predominately for mental and behavioural disorders due to acute stress reaction and adjustment disorders (25.3%), the use of alcohol (23.3%), major depressive disorder (10.1%), and mental and behavioural disorders due to the use of volatile solvents (7.8%).

### Pregnancy hospitalisations

Pregnancy-related hospitalisations for both prenatal and early childhood exposed groups were for delivery including caesarean sections and vacuum extractions (25.8%), other specified diseases complicating pregnancy (14.2%), medical abortion (11.5%), and false labour (7.3%).

### Infectious disease hospitalisations

Hospitalisations for infectious diseases in both prenatal and early childhood exposed groups were for intestinal issues (48.8%) including gastroenteritis, viral and other specified infections, viral infections (13.8%) and scabies (11.4%).

### Aboriginal and non-Aboriginal children

When stratified by Aboriginal status we found that Aboriginal children had a higher proportion of hospitalisations than non-Aboriginal children (Tables 4 and 5). Aboriginal children had an increased odds in hospitalisation for primary diagnoses relating to 12 ICD groups with non-Aboriginal children having an increased odds in eight. Aboriginal children exposed to FDV had a significant increased odds in hospitalisation for endocrinology disorders (OR 1.86; 95% CI 1.54–2.25), however, the non-Aboriginal exposed children had a non-significant increased odds (OR 1.15; 95% CI 0.77–1.72). Aboriginal children exposed also had higher odds of hospitalisation for infectious diseases than their non-Aboriginal counterparts (OR 1.76 95% CI 1.63–1.89; OR 1.22 95% CI 1.05–1.42 respectively). Comparing the groups where both Aboriginal and non-Aboriginal children both had increased odds, Aboriginal children had higher odds in all groups but two: pregnancy related admissions (OR 4.51; 95% CI 2.29–8.89; OR 1.82; 95% CI 1.45–2.28, respectively) and injury and poisoning (OR 1.57; 95% CI 1.41–1.75; OR 1.43; 95% CI 1.33–1.54, respectively).

**Table 4:** Adjusted* principal hospitalisations for non-Aboriginal children exposed and non-exposed to FDV in early childhood period.

ICD group	Non-Aboriginal children	Violence n = 2,618(%)	No violence n = 21,077(%)	OR (95%CI)	P-value
	Any hospitalisation <18 years	2,034 (77.7)	16,012 (75.97)	1.21 (1.09–1.34)	0.001
A00-B99	Infectious disorders	271 (10.4)	1,815 (8.61)	1.22 (1.05–1.42)	0.008
C00-D48	Neoplasms	32 (1.2)	369 (1.75)	0.92 (0.62–1.36)	0.665
D50- D89	Diseases of the blood ^^1^	13 (0.5)	118 (0.56)	0.80 (0.45–1.42)	0.439
E00-E89	Endocrinology disorders	23 (0.9)	204 (0.97)	1.08 (0.67–1.74)	0.75
F00-F99	Mental and behavioural disorders	29 (1.1)	226 (1.07)	2.32 (1.48–3.64)	<0.001
G00-G99	Diseases of the nervous system	117 (4.5)	860 (4.08)	0.90 (0.73–1.11)	0.307
H00-H59	Eye disorders	56 (2.1)	394 (1.87)	1.15 (0.84–1.57)	0.385
H60-H99	Ear disorders	148 (5.7)	1,469 (6.97)	0.89 (0.74–1.08)	0.242
I00-I99	Diseases of the circulatory system	25 (1)	174 (0.83)	1.47 (0.92–2.63)	0.107
J00-J99	Respiratory disorders	620 (23.7)	4,480 (21.26)	1.15 (1.03–1.27)	0.011
K00-K93	Digestive disorders	376 (14.4)	3,763 (17.85)	0.99 (0.87–1.12)	0.857
L00-L99	Dermatology disorders	117 (4.5)	754 (3.58)	1.45 (1.16–1.80)	0.001
M00-M99	Musculoskeletal disorders	60 (2.3)	584 (2.77)	1.13 (0.84–1.53)	0.405
N00-N99	Disease of genitourinary system	137 (5.2)	1,176 (5.58)	0.99 (0.81–1.21)	0.915
O00-O99	Pregnancy, childbirth and the puerperium ^^2^	10 (0.4)	126 (0.60)	4.51 (2.29–8.89)	<0.001
P00-P96	Certain conditions originating in the perinatal period	971 (37.1)	6,007 (28.50)	1.13 (1.03–1.25)	0.014
Q00-Q99	Congenital malformations, deformations and chromosomal abnormalities	163 (6.2)	1,318 (6.25)	0.88 (0.72–1.08)	0.198
R00-R99	Symptoms, signs and abnormal clinical and laboratory findings, not elsewhere classified	278 (10.6)	1,933 (9.2)	1.28 (1.10–1.48)	0.001
S00-T98	Injury, poisoning and certain other consequences of external causes (Injury and Poisons)	568 (21.7)	3,548 (16.8)	1.57 (1.41–1.75)	<0.001
Z00-Z99	Factors influencing health status and contact with health services	309 (11.8)	3,459 (16.4)	1.13 (0.98–1.30)	0.089

*Adjusted for sex, socioeconomic status, mothers maternal age, mother’s marital status, gestational age, disability, birth year, and remoteness.

^^1^ Firth bias correction applied due to quasi-complete separation caused by mother’s marital status

^^2^ Firth bias correction applied due to quasi-complete separation caused by birth year.

OR = Odds ratio, CI = Confidence Interval.

**Table 5:** Adjusted* principal hospitalisations for Aboriginal children exposed and non-exposed to FDV in early childhood period.

ICD group	All adjusted	Violence n = 4,400 (%)	No violence n = 20,919(%)	OR (95%CI)	P-value
	Any hospitalisation <18 years	3,926 (89.2)	17,965 (85.9)	1.45 (1.30–1.61)	<0.001
A00-B99	Infectious disorders	1,442 (32.8)	4,714 (22.5)	1.76 (1.63–1.89)	<0.001
C00-D48	Neoplasms	38 (0.9)	206 (1)	1.01 (0.70–1.45)	0.952
D50- D89	Diseases of the blood ^^1^	39 (0.9)	207 (1)	0.99 (0.70–1.39)	0.948
E00-E89	Endocrinology disorders	178 (4.1)	515 (2.5)	1.86 (1.54–2.25)	<0.001
F00-F99	Mental and behavioural disorders	135 (3.1)	458 (2.2)	2.71 (2.20–3.35)	<0.001
G00-G99	Diseases of the nervous system	161 (3.7)	738 (3.5)	0.96 (0.80–1.15)	0.653
H00-H59	Eye disorders	144 (3.3)	496 (2.4)	1.57 (1.28–1.92)	<0.001
H60-H99	Ear disorders)	742 (16.9)	2,433 (11.6)	1.55 (1.41–1.70)	<0.001
I00-I99	Diseases of the circulatory system	120 (2.7)	421 (2.0)	1.30 (1.05–1.62)	0.017
J00-J99	Respiratory disorders	1,981 (45.0)	7,792 (37.3)	1.47 (1.37–1.56)	<0.001
K00-K93	Digestive disorders	818 (18.6)	4,051 (19.4)	1.05 (0.96–1.15)	0.292
L00-L99	Dermatology disorders	945 (21.5)	3,095 (14.8)	1.61 (1.48–1.76)	<0.001
M00-M99	Muscular Skeletal disorders	200 (4.6)	882 (4.2)	1.17 (0.99–1.38)	0.069
N00-N99	Disease of genitourinary system	332 (7.6)	1,541 (7.4)	1.11 (0.97–1.26)	0.122
O00-O99	Pregnancy, childbirth and the puerperium ^^2^	118 (2.7)	838 (4.0)	1.82 (1.45–2.28)	<0.001
P00-P96	Certain conditions originating in the perinatal period	1,610 (36.6)	6,102 (29.2)	1.08 (1.0–1.27)	0.067
Q00-Q99	Congenital malformations, deformations and chromosomal abnormalities	241 (5.5)	972 (4.7)	0.98 (0.83–1.17)	0.805
R00-R99	Symptoms, signs and abnormal clinical and laboratory findings, not elsewhere classified	860 (19.6)	3,218 (15.4)	1.46 (1.33–1.60)	<0.001
S00-T98	Injury, poisoning and certain other consequences of external causes (Injury and Poisons)	1,410 (32.1)	5,713 (27.3)	1.43 (1.33–1.54)	<0.001
Z00-Z99	Factors influencing health status and contact with health services	886 (20.1)	4,337 (20.7)	1.30 (1.19–1.43)	<0.001

*Adjusted for sex, socioeconomic status, mothers maternal age, mother’s marital status, gestational age, disability, birth year, and remoteness.

^^1^ Firth bias correction applied due to quasi-complete separation caused by mother’s marital status

^^2^ Firth bias correction applied due to quasi-complete separation caused by birth year.

OR = Odds ratio, CI = Confidence Interval.

## Discussion

In our cohort study comprising of 49,953 children born 1987 to 2010 in WA we report that children exposed to FDV are more likely to be hospitalised than non-exposed children, even after adjustment for sociodemographic characteristics. This finding confirmed our hypothesis that FDV exposure in childhood is associated with a greater likelihood of childhood hospitalisation. Furthermore, as expected, we found that exposed children were more likely to be hospitalised for specific health issues such as mental health than non-exposed children.

### Demographics

Despite the initial matching of the cohort major differences were seen between the exposed and non-exposed groups in terms of Aboriginal status, early gestation (<37 weeks) and having a teenage mother. The reason for these differences may be multifactorial. It is acknowledged that FDV occurs in higher rates in Aboriginal than non-Aboriginal women [10, 32, 50, 51]. Additionally, children with teen mothers were over represented in the exposed group, this again could be partly explained by the over representation of Aboriginal children in the exposed group as Aboriginal teen mother rates are 5-times that of non-Aboriginal mothers [52]. However, a further possible explanation is that teen mothers are reported to be at greater risk of FDV than mothers over 19-years of age [53, 54]. The exposed children also had higher proportions of being born early gestation. While early gestation has been associated with prenatal FDV exposure [55], Aboriginal mothers are also more likely to deliver pre-term babies [56]. The multifactorial impact of ecological factors, including FDV, on early gestation is an area in need of further research.

### Hospitalisation

Children exposed to FDV had higher odds for hospitalisation and in multiple hospitalisation groups. Our findings are consistent with existing literature that suggests that environmental stressors play a major role in modifying physical and psychological health [57–59]. FDV exposure is considered to be one of the most prevalent stressors children can experience [17]. Exposure to FDV may disrupt adrenocortical activity with the exposed children experiencing heightened or lowered cortisol output [58, 60, 61], which has the potential to supress the immune system increasing the risk for physical and psychological disease [62]. The increased risk of diseases from the supressed immune system may be, in part, a possible explanation for the increased hospitalisation of children exposed to FDV.

A further possible explanation for the higher odds of hospitalisation is that the parents may not take the child to a regular medical practitioner for fear that FDV will be detected [63]. The lack of consistency in monitoring and treating chronic diseases may result in greater hospitalisations in categories such as ear disorders, dermatology disorders, respiratory disorders and endocrinology disorders. However, this explanation is only one possibility and further research is required. Further research is also required to investigate why the children had increased odds in some ICD groups but not all.

### Prenatal exposure

We found that children exposed to FDV in the prenatal period had greater odds of hospitalisation during childhood. A possible explanation for the increased hospitalisations could be attributed to the neuroendocrine effects of FDV exposure in utero where the stress experienced by the mother is thought to impact the foetus [64]. This stress impact has been acknowledged as a factor in negative birth outcomes of children exposed to FDV such as low birth weight and early gestation [65]. Further research on long-term health outcomes of children exposed to FDV in the prenatal period is required.

### Aboriginal children

Aboriginal children are disproportionately represented in our cohort, 62% of children in the exposed group were Aboriginal, a stark contrast to the 7% representation in the general child population in WA [66]. Aboriginal children exposed to FDV had higher odds of hospitalisation when directly compared to the odds in non-Aboriginal children. Furthermore, Aboriginal children had significant odds in endocrinology disorders, eye disorders, ear disorders, circulatory diseases and external factors influencing health. It is difficult to fully explain the differences seen in Aboriginal children’s hospitalisations. However, these results are likely to be related to the unmeasured contextual factors that Aboriginal children and their families may experience, including intergenerational trauma [19], attributed to the legacy of colonisation, including racism and forced removal of children [67] and an increased incidence of psychosocial stressors [68, 69]. Therefore, the results should be seen as an association between Aboriginal social factors and not necessarily a racial determinant. It is important that our findings are not used to stigmatise Aboriginal children and their families, they should be used as evidence for the need for prevention and early intervention strategies which are underpinned by Aboriginal communities’ cultural authority.

### Mental health

Children exposed to FDV in both the prenatal and early childhood period had a threefold increased odds of mental health hospitalisation. These results add to previous research that links children’s exposure to FDV and psychological health issues [8, 15, 16]. The main reason for mental health hospitalisations was acute stress reaction and adjustment disorders. Previous research by Bauer and colleagues [70] found an association with adjustment disorder in children whose mother experienced mental illness and FDV. However, they did not find an association when restricted to FDV alone. This discrepancy may be explained by the small number of mothers with FDV and not experiencing mental illness in the Bauer et al study (n = 69). Our datasets did not include maternal mental health service use and future research should include maternal mental health data to examine the effects of the co-occurrence on children.

Alcohol and solvent use was also a major factor for mental health hospitalisations in FDV exposed children. This externalised form of distress has previously been associated with exposure to FDV [71]. However, unlike Kilpatrick et al [71] who surveyed adolescents by phone, the children involved in our study were engaged in an inpatient service. Therefore, the hospitalisation of the child provides an opportunity to provide appropriate and evidence-based intervention, to the child and family, for not only the mental illness but for FDV.

### Pregnancy

We found a significant increase in odds of pregnancy-related hospitalisation in FDV exposed children. These findings suggest that children exposed to FDV are more likely to become pregnant in their childhood (< 18 years) than their non-exposed counterparts. These results align with previous findings which show that children exposed to FDV were more likely to become teen mothers [72]. Of concern, teenage mothers are at higher risk of FDV than older mothers thus perpetuating the risk for the exposed children and their children. Marked differences in pregnancy hospitalisations between Aboriginal and non-Aboriginal children could be attributed to the fact that Aboriginal mothers tend to be younger generally than non-Aboriginal mothers [73]. It has been highlighted that children exposed to FDV are more likely to experience FDV as adults [74], than those not exposed, continuing a cycle of abuse. This is an important area for prevention.

### Gastrointestinal problems

Children exposed to FDV in both prenatal and early childhood periods had higher odds of hospitalisation for infectious diseases, with gastrointestinal problems accounting for almost half of all hospitalisations. This is consistent with previous research which found that children exposed to FDV were more likely to have gastrointestinal problems [14]. However, these authors counted gastrointestinal problems as a psychosomatic issue whereas our gastrointestinal hospitalisations were mainly from gastroenteritis, viral and other specified intestinal infections. Our data does not contain the exact cause of the gastrointestinal hospitalisations and, therefore, we are unable to report on any psychosomatic component to the illness. Interestingly, despite the findings on gastrointestinal problems we did not find statistically significant odds for hospitalisation for digestive disorders. It is therefore important for future research to consider broad diagnosis groups when investigating gastrointestinal health outcomes of children.

### Injury and poisons

Importantly, we found an increased odds of hospitalisation for injury and poisoning for children exposed to FDV compared to non-exposed children, potentially explainable through co-occurrence of FDV and child abuse [75, 76]. Children exposed to FDV are at risk of injury from being caught in the path of physical assaults against their mother and being intentionally or ‘unintentionally’ hurt in the incident(s) [77, 78]. Furthermore, children living with FDV often live with multiple complex issues such as parental substance misuse, and parental depression and anxiety [79], with each of these issues potentially impacting on parenting capacity. Further research using linked administrative data from child protection services is required to investigate the child protection outcomes of children exposed to FDV and cooccurrence of health issues.

### Limitations

Some study limitations exist. Exposure to FDV was only captured in WA Police data where a male (with a domestic relationship to the mother) was charged, and in the hospital data when the child’s mother was admitted. Only 9% of Australian women seek support from police for FDV [4]. Of those who do seek support not all will result in a male being charged. Furthermore, the FDV captured in hospital data only counts women who are hospitalised and does not capture women who seek help from the Emergency Department or their General Practitioner. Therefore, our exposure is likely under-ascertained. Furthermore, due to the ‘endemic’ issue of FDV [4] it is possible that the non-exposed comparison group will contain children who are exposed to FDV but not documented in the available datasets, potentially biasing our results towards the null.

Due to the hidden nature of FDV we counted violence exposure at any point in prenatal period or the early childhood period. The child’s hospitalisation could have preceded or followed the recorded violence offence. However, as noted earlier, women’s help-seeking for FDV may be delayed and affected by the severity of abuse. Therefore, we counted all childhood hospitalisations during the respective prenatal period and early childhood period.

## Conclusion

Children exposed to FDV are more likely to be hospitalised than non-exposed children. Exposed children have a particular increased likelihood for mental health hospitalisation and pregnancy hospitalisation than non-exposed children. Within the exposed cohort differences were apparent between Aboriginal and non-Aboriginal children. Aboriginal children had greater odds for hospitalisation in the majority of ICD groups compared to their non-Aboriginal counterparts. These findings represent an important advance in the literature with respect to the burden of disease of children exposed to FDV and provide further support that children’s exposure to FDV as an area of public health concern. This study is the first of its kind. The use of linked administrative data has allowed us to investigate hospitalisations of children exposed to FDV at a population level as identified by hospital admissions and police incidents. The interaction of both the child and mother in health and/or police services provides an opportunity for appropriate and evidence-based intervention.
